# Maui-VIA: A User-Friendly Software for Visual Identification, Alignment, Correction, and Quantification of Gas Chromatography–Mass Spectrometry Data

**DOI:** 10.3389/fbioe.2014.00084

**Published:** 2015-01-21

**Authors:** P. Henning J. L. Kuich, Nils Hoffmann, Stefan Kempa

**Affiliations:** ^1^Integrative Proteomics and Metabolomics, Berlin Institute of Health, Berlin, Germany; ^2^Genome Informatics, Faculty of Technology, CeBiTec, Bielefeld University, Bielefeld, Germany; ^3^Integrative Proteomics and Metabolomics, Berlin Institute for Medical Systems Biology/Max Delbrück Center for Molecular Medicine, Berlin, Germany

**Keywords:** GC–MS, metabolomics, processing, software

## Abstract

A current bottleneck in GC–MS metabolomics is the processing of raw machine data into a final datamatrix that contains the quantities of identified metabolites in each sample. While there are many bioinformatics tools available to aid the initial steps of the process, their use requires both significant technical expertise and a subsequent manual validation of identifications and alignments if high data quality is desired. The manual validation is tedious and time consuming, becoming prohibitively so as sample numbers increase. We have, therefore, developed Maui-VIA, a solution based on a visual interface that allows experts and non-experts to simultaneously and quickly process, inspect, and correct large numbers of GC–MS samples. It allows for the visual inspection of identifications and alignments, facilitating a unique and, due to its visualization and keyboard shortcuts, very fast interaction with the data. Therefore, Maui-Via fills an important niche by (1) providing functionality that optimizes the component of data processing that is currently most labor intensive to save time and (2) lowering the threshold of expertise required to process GC–MS data. Maui-VIA projects are initiated with baseline-corrected raw data, peaklists, and a database of metabolite spectra and retention indices used for identification. It provides functionality for retention index calculation, a targeted library search, the visual annotation, alignment, correction interface, and metabolite quantification, as well as the export of the final datamatrix. The high quality of data produced by Maui-VIA is illustrated by its comparison to data attained manually by an expert using vendor software on a previously published dataset concerning the response of *Chlamydomonas reinhardtii* to salt stress. In conclusion, Maui-VIA provides the opportunity for fast, confident, and high-quality data processing validation of large numbers of GC–MS samples by non-experts.

## Introduction

Metabolomics is a field of study in which several different technologies are employed to identify and quantify metabolites in a given sample. Common technological platforms include nuclear magnetic resonance (NMR), liquid-chromatography mass-spectrometry (LC-MS), and gas chromatography–mass spectrometry (GC–MS). While the large variety of chemical characteristics of metabolites in biological samples is such that a complete metabolomic coverage, including lipids [a field of research on its own that has been termed lipidomics (Blanksby and Mitchell, 2010)], requires a combination of several technological platforms (Zhang et al., 2012). GC–MS technology combines high resolving power (Dunn et al., 2011), reproducibility (Pietzke et al., 2014), and a robust identification of a broad range of metabolite classes (Sumner et al., 2003).

One bottleneck in GC–MS metabolomics studies is the processing of raw machine data into a final datamatrix that contains metabolite abundances for every sample measured. Several processing steps are required, and usually include mass-specific baseline correction, smoothing, peak detection, retention index (RI) calculation, metabolite identification and alignment (IAA), and quantification. In addition to vendor software shipped with mass spectrometers, several freely available software packages exist to process GC–MS data, including, but not being limited to, XCMS (Smith et al., 2006), MzMine 2 (Pluskal et al., 2010), MetAlign (Lommen, 2009), TagFinder (Luedemann et al., 2008), Maltcms (Hoffmann et al., 2012, 2014), and others (Jonsson et al., 2005).

While these solutions allow for an automatized sample preprocessing, their use requires significant technical expertise and time to optimize parameters, which is often difficult to accomplish in biologically focused laboratories. Even after optimization of algorithm parameters, manual validation is nonetheless required for high-quality datasets and quickly becomes prohibitively time consuming as sample numbers increase (Coble and Fraga, 2014).

We have, therefore, developed a collection of software modules named VIA (Visual Identification and Alignment) that is aimed at providing a fast, user-friendly, and simultaneous manual inspection and correction of identifications and alignments of large numbers of GC–MS samples. It is based on Maui (Maltcms User Interface, http://maltcms.de/maui/index.html), a freely available, open-source, NetBeans Rich Client Platform-based GC–MS processing software. Maui-VIA also contains modules for RI calculation, a targeted library search for metabolite identification, metabolite quantification, and the export of the final datamatrix, which contains the user-validated metabolite abundances for every sample in the project. Maui-VIA, therefore, fills an essential niche to ensure dataset quality and validity irrespective of the software used for preprocessing steps, sample number, and user (software is available at http://bimsbstatic.mdc-berlin.de/kempa/software/kempaSoftware.html).

## Maui-VIA – Preprocessing of Data and Maui-VIA Setup

Depending on the MS vendor, a variety of acquisition and preprocessing software is available. ChromaTOF (version 4.50.8.0), the software shipped with the GC–TOF–MS used in this study (Leco-Pegasus III-TOF-MS-System, Leco), was used both for data acquisition and preprocessing. In the context of this paper, we will define preprocessing as the following steps as carried out in ChromaTOF: resampling of data (optional, sample reduction rate: 4, mass bins: 70–600, export: peg format), mass-specific baseline correction, smoothing, and peak detection (baseline offset: 1, data points used for smoothing: 13, peak width: 4, signal-to-noise ratio: 50, number of apexing masses: 2). Raw data and peaklists (required fields: Name, Retention Index, Area, UniqueMass, Quant Masses, R.T. (s), S/N, Integration Begin, Integration End, Full Width at Half Height) were then exported (netcdf and csv file formats, respectively). While Maui-VIA works fastest when using netcdf files, it is also possible to import raw files in the open mzML format (Martens et al., 2011). Peaklists should be converted to the csv file format exported by ChromaTOF and must contain the required fields mentioned above.

It is important to note that in addition to the samples of interest, a wash sample that only contains the alkane mixture used for the determination of RIs should be measured. The wash sample will later be used to facilitate the correct determination of alkane retention times in each sample to calculate peak-specific RIs.

Maui-VIA is based on the Netbeans Rich Client platform and is written in the Java programming language, making its use operating system independent. For its installation and execution, it is, therefore, necessary to install the Java Virtual Machine (JVM). Depending on the project size, processing in Maui-VIA might be memory intensive. It is, therefore, advised to allocate at least 4 gigabytes (GB) of RAM to the JVM, and at least 3 GB of RAM to Maui-VIA itself by modifying the –Xmx tag in its configuration file (*installationFolder*/Maui-VIA/etc/Maui-VIA.conf).

## Maui-VIA Metabolite Databases

Metabolite databases that can be imported into Maui-VIA are in the text-based msp format that was developed by the National Institute for Standards and Technology. The Lib2NIST and NIST MS Search programs are freely available and are able to convert and export into msp format (http://chemdata.nist.gov/mass-spc/ms-search/, http://chemdata.nist.gov/mass-spc/ms-search/Library_conversion_tool.html).

In addition to the msp format requirements, Maui-VIA requires the metabolite database entries to contain the metabolite name, derivatization information, derivatization product, retention index preceded by “RI:”, and the name of the database, separated by underscores. A specific example would be: Phenylalanine_(1TMS)_BP_RI:1551_pubDB.

The entries must be unique and contain all five elements. The database library that was used to analyze the *Chlamydomonas reinhardtii* dataset described in this paper, containing correctly structured metabolite entries, is available online.

## Maui-VIA Project Setup, Data Import, and Metabolite Database Conversion

A new project is created with the baseline-corrected raw files (netcdf or mzML format) of a measurement batch. All files need to be associated with a “treatment” attribute that can be used to group related samples. Once a project is successfully created in the form of a db4o database (https://source.db4o.com/db4o), the user needs to import a peaklist in ChromaTOF format for each sample as well as a metabolite database in msp format that will later be used to identify metabolites in the samples. All functionality is accessible by a right-click on the project and traversing the menus displayed. The metabolite database needs to be converted within Maui-VIA for subsequent visualization.

While the previous steps complete the required project setup, the user is provided with the option of loading a file specifying quantification masses for metabolites present in the provided database. The file should contain one line per metabolite identifier in the provided database, each line containing the full metabolite database entry name and the desired quantification mass/es (for multiple masses, separated by semicolons), separated by tabs. An example file is available online.

For every database entry for which quantification masses are not specified in the file, or in the case of the user providing no quantification mass file, the quantification will be performed using the five masses with highest intensity of the library spectrum, excluding all masses below 85 and the masses 147 and 148, latter of which result from the trimethylsilyl (TMS) derivatization and are, therefore, not metabolite specific.

## Maui-VIA Retention Index Determination

The user is required to provide the number of alkanes present in the mixture used in the project as well as their approximate retention time in the wash sample. This information is used to identify the alkane peaks in the wash by searching for the peaks with maximal intensity considering only the alkane-specific masses provided by the user (for electron ionization, for example, the masses 71, 85, and 99). These peaks are matched by calculating the minimal difference between candidate peak and provided alkane retention times. The alkane peaks identified in the wash are used to produce a “wash database,” containing the spectra and retention times of the alkanes identified in the wash.

Maui-VIA then performs a targeted search for the alkanes in all samples by comparing the wash database alkane spectra and peak spectra found within a time window of ±15 s in each sample. Spectral comparisons are scored throughout Maui-VIA by a cosine similarity score between the two mass spectrum vectors *A* and *B*, and are calculated as follows:

$$\mathrm{similarity}=\frac{\sum_{i=1}^{n}A_{i}\;x\;B_{i}}{\sqrt{\sum_{i=1}^{n}{\left(A_{i}\right)}^{2}\;}\;x\;\sqrt{\sum_{i=1}^{n}{\left(B_{i}\right)}^{2}}}$$

This similarity score is multiplied by a factor of 1000 to obtain a more convenient number format. The peaks that were assigned the highest similarity score are putatively assigned the alkane names and are subsequently visualized in the Maui-VIA IAA interface, where they can be inspected and, if necessary, corrected. Once all alkanes have been assigned in all samples, RIs of all peaks in all samples are calculated by linear interpolation (Kovats, 1958; van Den Dool and Kratz, 1963; Strehmel et al., 2008).

## Maui-VIA Reverse Search

After the RI correction has been applied to the project, the user is ready to identify metabolites in the samples of interest. There are two principle ways of putatively identifying peaks in a sample: (1) every peak in every sample could be compared to entries in a metabolite database and, if a match is found, be assigned an identity (here termed “forward search,” the strategy employed by ChromaTOF) or (2) a database of metabolites of interest can be used to search for the presence of its entries in the samples of interest (here termed “reverse search”).

One complication of the first strategy is the possibility of assigning a single metabolite identifier to multiple peaks in a single sample. False positives in this untargeted search are theoretically minimized by setting a minimum similarity score required for putative identification; however, experience has shown that setting a single threshold for a broad range of metabolites is difficult since an appropriate threshold for a metabolite of interest is likely either too stringent or insufficiently stringent for many others. Striking a compromise results in labor-intensive inspection and correction of great numbers of both false positives and false negatives.

Maui-VIA, therefore, employs a reverse search (RS). All peaks within an RI window of ±13 RI units of each sample are compared to the respective entries in the provided metabolite database. The comparisons are ranked by the cosine similarity score penalized for large RI deviations (see Table 1). The peak with the highest score is putatively identified, provided that its similarity score is greater than the threshold score of 800. Thus, at most one peak is assigned a metabolite identifier per sample, while the score threshold ensures a low rate of false positives for most database entries.

**Table 1:** **Similarity score penalties based on RI difference of peaks to database entries**.

RI difference (RI units)	<1.5	>1.5	>3	>4	>5
		<3	<4	<5	
Score penalty (%)	0	3	5	7	15

Once the RS is completed, putative identifications are assigned and can be inspected and, if necessary, corrected using the IAA interface.

## Maui-VIA Visual Identification and Alignment Interface

**Figure 1:**
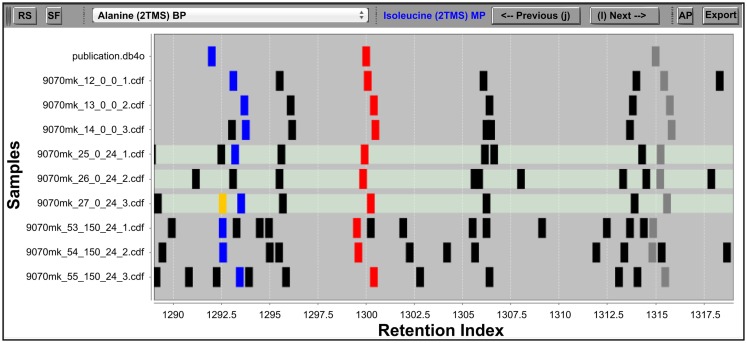
**The IAA RIView**. The “RS” (reverse search) button initiates the RS. The “SF” (save fusions) button saves fusions made in the IAA into the project database. The dropdown list in the top middle allows the user to directly jump to a metabolite database entry of interest, while the “Previous” and “Next” buttons (also accessible via keyboard shortcuts as indicated on the buttons) serve to traverse the database one entry at a time. The “AP” button opens the MSView, and the “Export” button exports accepted peak groups into a database container. The RI chart *y-*axis is populated by the metabolite database followed by rows of samples, and peaks abstracted into vertical bars are plotted along the *x*-axis according to their RI. Differently colored peaks indicate different stages of processing: unknown peaks are black, putatively identified peaks are red, the library entry currently selected and its corresponding putative identifications in the samples are colored blue, while the horizontal traversal within a sample as controlled by the MSView is indicated by orange. The three highlighted rows of samples are displayed in the MSView.

**Figure 2:**
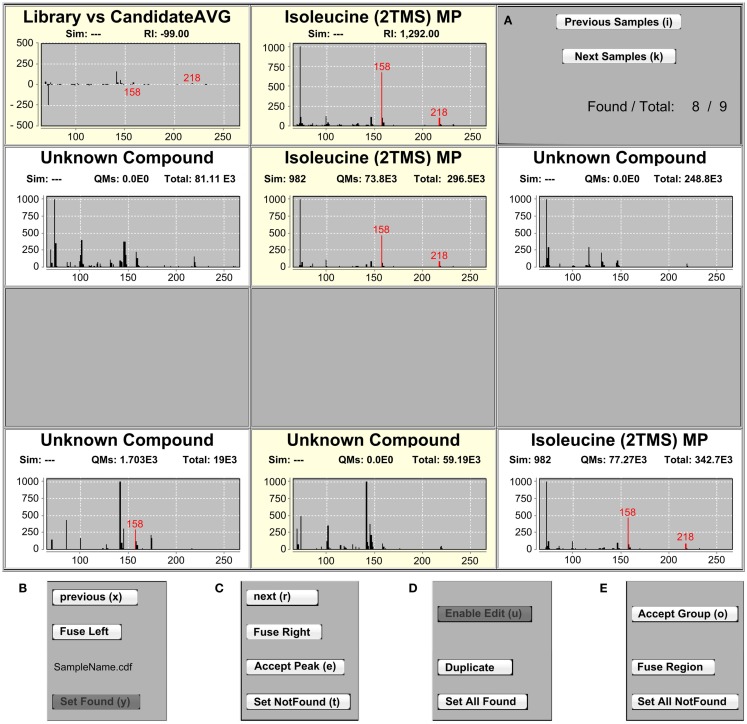
**The IAA MSView**. **(A)** The MSView is composed of four rows of spectra and buttons that are accessible via keyboard shortcuts. The chart in the middle of the top row displays the mass spectrum of the currently selected library entry. The chart to the left shows the spectrum difference between the library entry and putative assignments in the samples. Putatively assigned metabolites in samples are displayed in the middle chart of the three rows beneath, the charts to their left and right displaying the mass spectra of the peaks to the immediate left and right, respectively. **(B,C)** The samples can be traversed horizontally by the “Previous” and “Next” buttons of each row as well as vertically by the “Previous Samples” and “Next Samples” buttons in the right panel of the top row. Assignments of peaks in a sample can be removed by the “Set NotFound,” and added by the “Set Found” buttons, respectively. Peaks can be fused with their right or left neighbor using the “Fuse Left” and “Fuse Right” buttons in each row. “Accept Peak” leads to the assignment of the library identifier to the currently selected peak and recalculates the similarity score. **(D,E)** Peaks containing a combined spectrum of co-eluting metabolites can be duplicated with the “Duplicate” button. For convenience, it is possible to add or remove peak assignments to/from all samples at once with the “Set AllFound” and “Set AllNotFound” buttons, as well as to fuse entire regions of peaks with the “Fuse Region” button. Once identifications and alignments are confirmed, “Accept Group” locks the selection from further editing and marks the identifications to be ready for export.

The Maui-VIA IAA interface is composed of two windows, each of which displays one of the two principle pieces of information obtained from GC–MS methods to identify metabolites. The RIView window displays the distribution of peaks in a sample according to their RI and, therefore, visualizes GC retention time (Figure 1). The MSView visualizes peak spectra, therefore representing the information obtained by the MS (Figure 2A). Both the RIView and MSView visualizations are based on the Java charting library JFreeChart (http://www.jfreechart.org/).

The IAA was designed with the intention of allowing the visually guided alignment and annotation of a large number of samples at the same time to allow for a fast recognition of IAA errors. Alignment, in the context of this paper, does not result from an initial grouping of features that are then collectively identified. Rather, the RS putatively identifies metabolites in each sample individually, which simultaneously produces a putative alignment. Both can then be inspected and corrected using the IAA.

The crucial difference to most other visualizations of GC–MS data is the abstraction of chromatograms into a binary representation. Each peak detected in a sample is represented by a vertical bar, whose size is independent of the underlying peak shape and size, and whose position is specified by the peak apex RI (Figure 1). This simplification allows a user to immediately grasp the structure of acquired data, and alignments of peak groups become immediately obvious. The first row of the chart is always occupied by the database whose metabolite entries were searched for in the samples, followed by the samples themselves. The database entries can be traversed by the “previous” and “next” buttons of the RIView.

The three rows highlighted in the RIView are represented in the MSView, which is composed of four rows of mass spectrum charts (Figure 2A). The first row displays the spectrum of the currently selected library peak in the middle panel as well as the difference between all spectra of currently annotated sample peaks and the library spectrum in the left panel. Quantification masses for the current metabolite are indicated in red in all spectra. In the right panel, two buttons can be used to traverse the samples vertically (indicated by the highlighting in the RIView) and a label displays the number of currently annotated samples for the selected library metabolite (Figure 2A).

The three rows below represent the highlighted sample rows in the RIView. The middle spectrum of each row corresponds to the spectrum of the currently selected peak in the corresponding sample (colored blue in the RIView; putative identifications of other library entries are red; already corrected and confirmed identifications are gray or green if currently selected), while the spectrum panels to the left and right represent the spectra of those peaks that are immediately to its left and right, respectively. Spectrum charts also include information on the cosine similarity score of each peak calculated by the RS as well as the quantification mass specific and total intensities of each peak. The buttons “previous” and “next” (Figures 2B,C) in each row allow for the horizontal traversal of samples, leading to updates of the mass spectrum charts in the MSView (Figure 2A, fourth row) and the RIView peak coloring (selected peak orange, previous putative identification remains blue, Figure 1, lowest highlighted row). Samples in which the database entry is not annotated appear blank in the MSView and have no blue or orange peak in the RIView (Figure 1, highlighted middle row, Figure 2A, third row). The database as well as vertical and horizontal sample traversal functions allows for the fast exploration of measurement batches in the excess of 100 samples.

The second intention was to provide the ability to quickly correct IAA errors to ensure validity and expert-level data quality. Inspection and correction speed are facilitated by all functions being accessible by keyboard shortcuts. Vertical traversal allows for the fast inspection of putative identifications. Horizontal traversal allows for the fast scanning of surrounding peaks for each sample if the putative identification appears dubious. The functions “Set Found” and “Set NotFound” allow the user to add and remove putative identifications from samples, respectively (Figures 2B,C). The “Fuse Left” and “Fuse Right” functions allow the user to fuse the currently selected peak with the peak to the immediate left or right, respectively (Figures 2B,C). The fusion simply recalculates the peak RI to be the average of the two fused peaks, and sets the new peak start and end scans to the first and last scan of the two fused peaks, respectively. This function is useful in cases of incorrect peak deconvolution that occur in ChromaTOF, where masses belonging to a single peak are split into two separate peaks. For the reverse case, in which a single peak contains two co-eluting metabolites, the “Duplicate” function is available (Figure 2D). It duplicates all currently selected (blue in RIView) peaks, so that one copy can be assigned to one of the two co-eluting metabolites each. Please note that this is only valid if the metabolites do not share any quantification masses. The “Accept Peak” button for each row recalculates the similarity score for the currently selected peak without the RI penalty used in the RS and assigns the currently selected database identifier (Figure 2C). For convenience, the functions “Set AllFound” and “Set AllNotFound” enable the user to add or remove putative identifications to/from all samples if it is obvious that the RS has failed due to insufficient peak quality and/or intensity (Figures 2D,E). Similarly, “Fuse Region” allows the fusion of peaks across all samples in a common, user-specified RI window with a single function (Figure 2E). Once a library metabolite is deemed correct, “Accept Group” locks the annotation, meaning that the peaks have been validated, should not be changed, and are ready to be exported (Figure 2E). “Enable Edit” removes the lock, and makes peak groups accessible again (Figure 2D). In combination, these functions allow the fast traversal of the metabolite database one metabolite at a time, as well as the inspection and correction of putative identifications of the RS in all samples (please note that this is most efficiently and conveniently achieved by the use of two monitors, each displaying the RIView or MSView).

Finally, the “Export” button in the RIView exports all accepted peak groups into a peak group container (named “samplesPeak- Groups”) (in the case of the RI calculation module, “Export” calculates RIs for all peaks in all samples).

If the user’s methodological setup includes the addition of a normalization standard to be able to correct for sample preparation and injection differences, she simply needs to include the standard in the metabolite database provided and process it like any other metabolite identification. The normalization of metabolite intensities of each sample can then be carried out after data export.

## Maui-VIA Metabolite Quantification and Export

Once the peak group container has been exported, the user can quantify the metabolites in each sample. Since there is no perfect rule for the choice of quantification masses, the default strategy was chosen to be the use of the five most abundant masses in the library metabolite spectrum. This has proven to be a good compromise between a robust quantification ensured by considering the abundance of several fragments of a metabolite, and a specific quantification ensured by choosing masses that are not likely shared by co-eluting metabolites. Since quantification mass specificity cannot be guaranteed by any general strategy and is dependent on the analyzed samples as well as the metabolites of interest (Lisec et al., 2006), Maui-VIA allows the user to specify quantification masses for every database entry to ensure quantification specificity. A reasonable strategy, therefore, would be to use the default of the five most abundant masses for most metabolites and specifying quantification masses only for those known to have co-eluting metabolites with identical masses.

Quantification is performed by collecting all scans of a peak as defined by its peak boundaries determined by the peak calling algorithm employed during preprocessing from the baseline-corrected raw data files used to initiate the project. The sums of the intensities of each quantification mass of all scans are summed, leading to the determination of the summed peak area for all quantification masses. This information is saved in the peak group container. One advantage of this is that if it becomes apparent that some quantification masses were poorly chosen, the user can simply delete the peak group container, reload an updated version of the quantification masses file, re-export the peak group container, and repeat the quantification step.

In order to export the final datamatrix, a right-click on the peak group container offers an export option, followed by an option of specifying a folder name. Two datamatrices are exported in csv format, one containing the raw area peak values (“rawAreaMatrix.csv” – quantification of the peak detection software if provided in the initially imported peaklists in the “Area” field), and the area peak values determined by Maui-VIA (“AreaMatrix.csv”).

## Maui-VIA *Chlamydomonas reinhardtii* Use Case

To demonstrate the validity of the sample annotation and quantification using Maui-VIA, nine samples of a previously published *C. reinhardtii* dataset (Mastrobuoni et al., 2012) were processed manually by an expert in ChromaTOF, and the result compared to the same samples analyzed with Maui-VIA. The dataset containing baseline-corrected raw files and peaklists, an example metabolite database of nine metabolites, and a quantification mass specification file is available online.

The dataset is composed of three biological replicates of three conditions: (1) samples taken at the initial time point before the application of a salt stress, (2) samples taken after a continuous 150 mM sodium chloride salt stress for 24 h, and (3) control samples taken after 24 h without salt stress.

**Figure 3:**
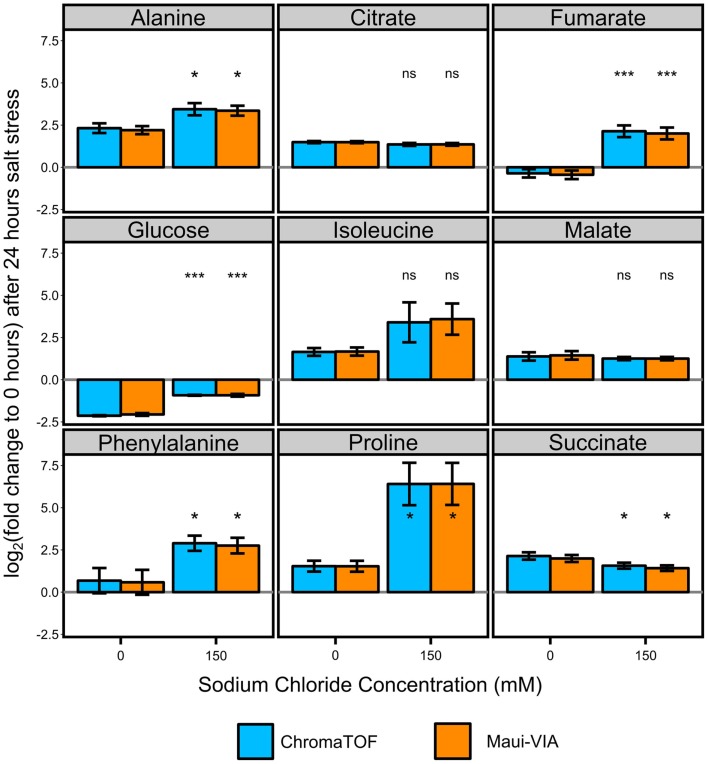
**Metabolite responses to salt stress in *Chlamydomonas reinhardtii* are indistinguishable when comparing Maui-VIA with ChromaTOF**. Statistical differences in amino acids (alanine, isoleucine, phenylalanine, and proline), tricarboxylic acid cyclic intermediates (citrate, fumarate, malate, succinate), and glucose between the salt stress (150) and control condition (0) are identical, independent of the software used (light blue = ChromaTOF, orange = Maui-VIA). Metabolite levels are plotted as the mean of the log_2_ transformation of the fold change of the presented conditions to the untreated control time point of 0 h. (*t*-test, FDR-corrected, ns, not significant, *p-value <0.05, ***p-value <0.01, error bars display 95% confidence intervals, *n*  = 3).

**Figure 4:**
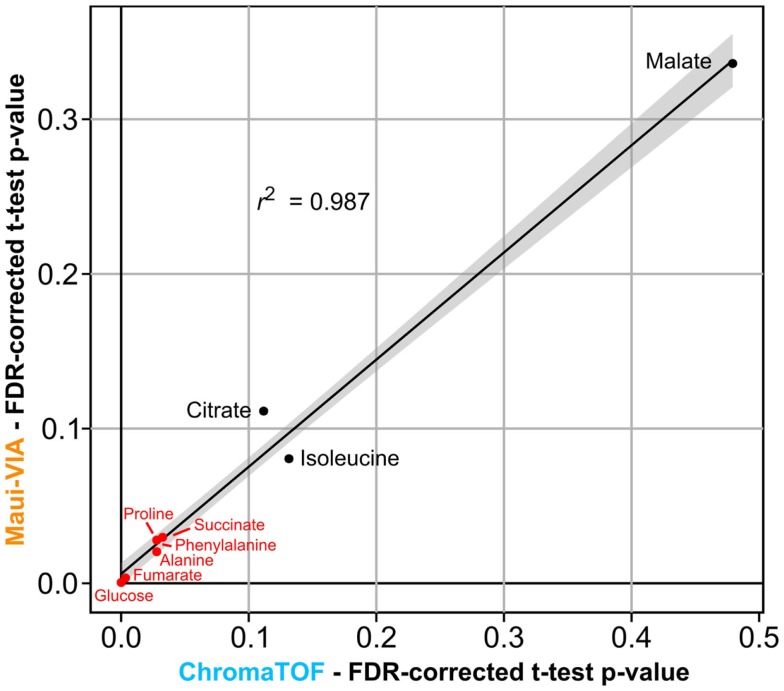
***p*-values of metabolite differences obtained with Maui-VIA and ChromaTOF strongly correlate**. FDR-corrected *p-*values obtained for each metabolite are plotted for Maui-VIA and ChromaTOF on the *y*-axis and *x*-axis, respectively. *p-*values indicating statistical significance are indicated in red, others in black. The *r*^2^ value of the linear regression displayed is 0.987.

The samples were normalized to the internal normalization standard ^13^C-sorbitol. When plotting the log2 transformed value of the fold change of the control and treatment samples after 24 h to the initial condition, it is evident that the data obtained would lead to identical conclusions independent of the software used for analysis and metabolite class investigated (Figure 3). The minimal differences likely result from the different quantification strategies applied. Importantly, independent of the software used for analysis, *t*-tests for all metabolites displayed in Figure 3 proved to be qualitatively identical [statistically significant at α = 0.05, false discovery rate (FDR)-corrected (Benjamini and Hochberg, 1995)], and the *p*-values themselves were strongly correlated (Figure 4).

## Conclusion

Maui-VIA solves two principle issues currently pervasive to GC–MS-based metabolomics research: (1) The time cost associated with the production of validated, high-quality datasets of many samples and metabolites and (2) the difficulty for non-experts to process GC–MS data efficiently without requiring extensive training to produce meaningful information. In our hands and depending on project size and desired depth of analysis, Maui-VIA has decreased processing time by around 5- to 10-fold. In general, the more samples are contained in a project, the more time is saved in comparison to other manual validation methods. Furthermore and crucially important, its interface makes GC–MS data processing seem intuitive even to complete novices, who have been able to reproduce entire validated datasets in the excess of 50 samples annotated with at least 80 metabolites within a week of beginning their metabolomics careers in our laboratory.

It is our hope that Maui-VIA will be helpful to experts by vastly decreasing processing time in the face of ever growing experimental complexity and non-experts by making GC–MS technology more accessible and intuitive.

## Conflict of Interest Statement

The authors declare that the research was conducted in the absence of any commercial or financial relationships that could be construed as a potential conflict of interest.
